# Protective Effect and Mechanisms of Radix Astragali Injection on the Intestinal Mucosa of Rats with Obstructive Jaundice

**DOI:** 10.1155/2010/757191

**Published:** 2010-03-14

**Authors:** Zhang Xiping, Weng Ke, Yu Yaping, Zhao Hongchan, Cheng Qihui

**Affiliations:** ^1^Department of General Surgery, Hangzhou First People's Hospital, Zhejiang Province, Hangzhou 310006, China; ^2^College of Clinical Medicine, Zhejiang Traditional Chinese Medicine University, Zhejiang Province, Hangzhou 310053, China; ^3^Department of Testing, Hangzhou First People's Hospital, Zhejiang Province, Hangzhou 310006, China; ^4^Department of Gynaecology and Obstetrics, Hangzhou First People's Hospital, Zhejiang Province, Hangzhou 310006, China

## Abstract

*Objective*. To research the protective effects and mechanisms of Radix Astragali injection on the intestinal mucosa of rats with obstructive jaundice (OJ). *Methods*. The rats were randomly divided into sham-operated, model control and Radix Astragali treated group. We observed the pathological changes of intestinal mucosa, expression levels of Bax and NF-*κ*B proteins, and apoptosis indexes in intestinal mucosa as well as serum NO, MDA and SOD contents, respectively, on 7d, 14d, 21d and 28d after operation. *Results*. The pathological severity score (on 7d and 14d), apoptotic indexes (on 14d) of the intestinal mucosa and serum MDA content (on 14d) of treated group were significantly lower than those in the model control group (*P* < .05). The serum SOD contents (on all time points) of treated group were significantly higher than those in the model control group (*P* < .05). The sham-operated group (on 21d) of the product of staining intensity and positive rate of Bax protein was significantly lower than model control group (*P* < .05). *Conclusion*. Radix Astragali injection could protect the intestinal mucosa of OJ rats by increasing the content of SOD, reducing the content of MDA, inhibiting the apoptosis and relieving the pathological changes of intestinal mucosa.

## 1. Introduction

During obstructive jaundice, the impairment of intestinal mucosal barrier and the translocation of endotoxins and intestinal bacteria can cause intestinal infections and secondary multiple organ injury, which are pivotal for the deterioration of the disease [1–6]. Therefore, it is of great significance to protect the small intestinal mucosa [7]. OJ is sensitive to auxiliary treatment with traditional Chinese medicine. Astragalus injection, as an extract from Radix Astragali, is characterized by low cost, extensive pharmacological effects and few side effects and therefore has unique advantages and prospects in the treatment of OJ. Astragalus injection is one of the famous injections of traditional Chinese medicine. Astragalus injection contains polysaccharide, saponin, flavones, and trace elements [8]. Although some studies [8, 9] showed that Astragalus could significantly improve the oxygen free radical-scavenging abilities, inhibit excessive inflammatory response, and thereby mitigate hepatic and renal injury in OJ rats, no study on the protective effects of astragalus on intestinal mucosa was found in domestic and foreign literature. In this study, we investigated the protective effects and mechanisms of Astragalus injection on small intestinal mucosa of OJ rats to provide a theoretical basis for clinical application of this injection.

## 2. Materials and Methods

### 2.1. Materials

The healthy male SD rats of clean grade, weighing between 270 and 330 g, were provided by the Laboratory Animal Research Center of the Zhejiang University of Traditional Chinese Medicine (Hangzhou, China). The sodium pentobarbital was purchased from Sigma Corporation (Sigma-Aldrich, St. Louis, MO, USA). Radix Astragali injection (each 10 mL vial contains active components equivalent to 20 g of the original medicine) was purchased from Chiatai Qingchunbao Pharmaceutical Co., Ltd (Hangzhou, China). The serum nitrogen monoxidum (NO), malonaldehyde (MDA), and superoxide dismutase (SOD) kits were all purchased from Nanjing Jiancheng Bioengineering Research Institute (Nanjing, China), and the calculation units for content are respectively *μ*mol/L, nmol/mL, and U/mL. The anti-NF-*κ*B P65 and anti-Bax antibody were purchased from Santa Cruz Biotechnology, Inc. (Santa Cruz, California, USA) TUNEL assay kit was purchased from Takara Bio Inc. (Jingdu, Japan).

### 2.2. Methods

#### 2.2.1. Animal Grouping and Preparation of OJ Models

180 rats were utilized for OJ-associated experiments and randomly divided into sham-operated, model control, and treated group (*n* = 60), which were further randomly subdivided into 7d, 14d, 21d, and 28d groups (*n* = 15) according to time duration after operation. After rats were anesthetized with an intraperitoneal injection of 2.5% sodium pentobarbital (0.2 mL/100 g), the abdominal cavity was opened to identify and dissociate common bile duct along the hepatoduodenal ligament. For rats in the model control group and the treated group, the proximal end of common bile duct was double ligated with surgical threads, common bile duct was cut off, and a layered suture of the abdominal wall was performed to close the abdominal cavity. For rats in the sham-operated groups, common bile duct was only dissociated but not ligated, and a layered suture of the abdominal wall was also performed to close the abdominal cavity. An intraperitoneal injection of Radix Astragali injection at a dose of 0.75 mL/100 g/d was given to rats in the treated groups while equal volume of physiological saline solution was used in the sham-operated and the model control group. Different injection was maintained until the end of the 7-day, 14-day, 21-day, and 28-day observation period in the corresponding groups [8].

#### 2.2.2. Determination of Experimental Parameters

The mortality rate of rats in each group was recorded. After mercy killing rats anesthetized by sodium pentobarbital in batches, we collected the serum of rats to detect NO, MDA and SOD contents and observed the pathological changes of intestinal muscoa. The determination of these serum parameters was conducted according to the instructions provided by the kits. The pathological severity score of intestinal muscoa was conducted according to our report for related standard. And then we prepared the tissue microarrys section for intestine tissue with diameter of 1.5 mm, stained them, observed the changes in the expression levels of Bax and NF-*κ*B P65 proteins as well as the apoptosis index of intestinal muscoa.

#### 2.2.3. Immunohistochemical Staining of Bax and NF-*κ*B P65 Proteins in the Intestinal Muscoa

Envision two-step method was used to detect the expression levels of Bax and NF-*κ*B P65 protein in the intestinal muscoa. The evaluation standard was as follows: (1) the staining intensity was evaluated according to the extent of cell coloration: “−” represented negative staining; “+” represented mild staining, positively stained cells showed a yellow pigment; “++” represented moderate staining, positively stained cells showed a brown pigment; “+++” represented intense staining, positively stained cells showed a dark brown pigment, each of which was scored as 0, 1, 2, and 3 points, respectively, during statistical analysis; (2). the evaluation standard of the positive rate: there was no positive cells (−); the percentage of positive cells was less than 25% (+); the percentage of positive cells ranged between 26% and 50% (++);the percentage of positive cells was more than 50% (+++), each of which was scored as 0, 1, 2, and 3 points, respectively, during statistical analysis.

#### 2.2.4. TUNEL Staining in the Intestinal Muscoa

In tissue microarrays sections, DNA nick in situ end-labeling (TUNEL) staining steps were performed as follows: baking sections under 60°C for 30 minutes, routine deparaffinage, and Milli-Q wash for 5 minutes. Processing tissue with Protease K (10 ug/uL) under room temperature for 15 minutes, PBS washes for 5 minutes. Using 3% H_2_O_2_ solution to block endogenous peroxydase for 5 minutes, PBS wash for 5 minutes×twice. Adding 30 uL reaction solution in freezing condition (TdT Enzyme: Labeling Safe Buffer = 1  : 10), 37°C incubation for 90 minutes, PBS wash for 5 minutes × twice. Adding 50 ul Anti-FITC HRP Conjugate, 37°C incubation for 30 minutes, PBS wash for 5 minutes × twice. DAB coloration, Milli-Q wash to terminate coloration. Hematoxylin counterstain, water wash and wash fully with water after differentiation till return blue; routine dehydration and transparence; neutral gum mounting. The apoptotic index was calculated. Apoptotic index = apoptotic cell count/total cell count × 100%.

#### 2.2.5. Statistical Analysis

After input into the Excel sheets, the compiled data was read into SPSS15.0 for further analysis. Normal data was expressed as means (standard deviation) while nonnormal data were expressed as medians (interquartile range). Analysis of variance and pairwise comparisons were used in normal data; whereas nonnormal data were subjected to nonparametric test, among which Kruskal-Wallis *H* test was used for pairwise comparisons and Mann-Whitney *U* test for multiple comparisons. Yates' chi-square test (*χ* ^2^) was used for intergroup comparisons of mortality rates.

## 3. Results

### 3.1. Comparison of Mortality Rate

All rats were alive in the sham-operated groups on all time points after operation. 2 and 1 rats died in the model control and treated group on 7d, respectively; 4 and 3 rats died in the model control and treated group on 14d, respectively; 4 rats died in both model control and treated group on 21d; 7 and 6 rats died in the model control and treated group on 28d, respectively. The total mortality rate of the model control and treated group on 28d were significantly higher than those in the sham-operated group (*P* < .001). There was no marked difference between the model control and treated group (*P* > .05).

### 3.2. Pathological Changes in Intestinal Mucosa

#### 3.2.1. Sham-Operated Group

**Gross Pathological Changes**

No obvious abnormality was seen.

**Pathological Changes under Light Microscopy**

**Figure 1:**
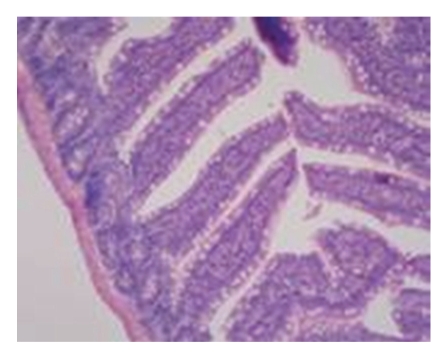
Sham-operated group-28d mucous membrane of small intestine is approximately normal HE × 100.

No obvious difference in pathological changes was observed among each time point after operation. The intestinal mucosa was normal in the majority of rats. The intestinal mucosal epithelium was not intact and inflammatory cell infiltration of proper layer was seen in very few rats (see Figure 1).

#### 3.2.2. Model Control Group

**Gross Pathological Changes**

On 7d after operation, intestinal wall and peritoneum became jaundice in the majority of rats. On 14d after operation, varying degrees of yellow staining of the intestinal wall and peritoneum were seen in the majority of rats. The intestinal canal was enlarged and showed fluid retention. On 21 and 28d after operation, yellow staining of the intestinal wall and peritoneum were seen in all rats.

**Pathological Changes under Light Microscopy**

**Figure 2:**
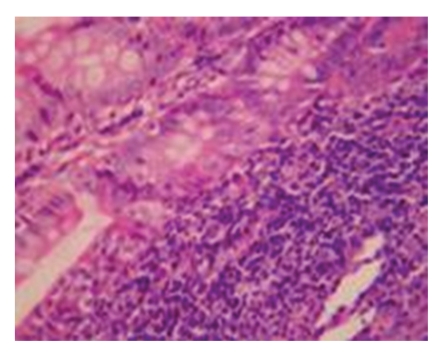
Model control group-28d a large inflammatory cells infiltration in small intestinal wall HE × 200.

**Figure 3:**
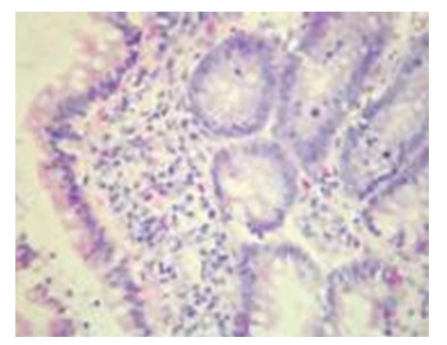
Model control group-28d a small quantity acidophile cells infiltration in intestinal lamina propria HE × 200.

No obvious difference in pathological changes was observed among each time point after operation. On 7d after operation, intestinal mucosa was normal in the majority of rats, and the edema of submucous layer were present in very few rats. On 14d after operation, intestinal mucosa was normal in the majority of rats but not intact in some rats, and the edema of proper layer, submucous layer, and serosal layer were seen in some rats. On 21d after operation, intestinal mucosa was not intact in the majority of rats, the edema of proper layer, submucous layer, and serosal layer were seen in the majority of rats, and very few rats showed no abnormality of the intestinal mucosa. On 28d after operation, focal necrosis in intestinal mucosal epithelium as well as the edema of proper layer, submucous layer and serosal layer were seen in the majority of rats (see Figures 2 and 3).

#### 3.2.3. Treated Group

**Gross Pathological Changes**

On 7d after operation, no obvious difference was observed when compared to that in model control group. On 14d after operation, intestinal wall became jaundice in half of the rats, but intestinal canal was not enlarged and showed no fluid retention. On 21, and 28d after operation, no obvious difference was observed when compared to those in model control group.

**Pathological Changes under Light Microscopy**

**Figure 4:**
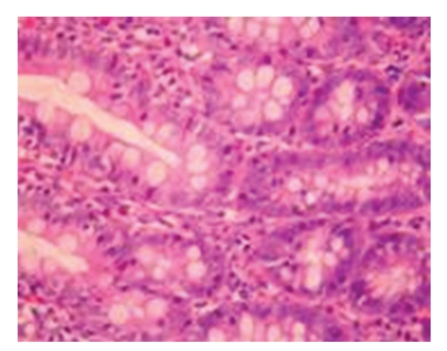
Treated group-28d mucous membrane of small intestine is approximately normal HE × 200.

The pathological changes in the small intestine of rats at various time points showed varying degrees of mitigation. On day 7 in treated group, the small intestine of the majority of rats showed no abnormality while the small intestine of extremely few rats showed inflammatory cell infiltration in proper layer, submucous layer, and serosal layer. On day 14 in treated group, the small intestine of some rats showed no abnormality, the small intestine of some rats showed inflammatory cell infiltration in proper layer, submucous layer, and serosal layer, and the small intestine of extremely few rats showed focal necrosis in the mucosa. On day 21 in treated group, the small intestine of some rats showed no abnormality while the other rats showed inflammatory cell infiltration in proper layer, submucous layer and serosal layer. On day 28 in treated group, the small intestine of some rats showed inflammatory cell infiltration in proper layer, submucous layer and serosal layer while the extremely few rats showed no abnormality (see Figure 4).

### 3.3. Comparison of the Pathological Severity Scores

The pathological score standard of intestinal mucosa was referred the report [10]. Pathological severity scores of the sham-operated group (on 7, 14, and 21d) was significantly lower than model control group (*P* < .05). The sham-operated group (on 7d) were significantly lower than treated group (*P* < .05). The scores in treated group (on 7 and 14d) were significantly lower than those in model control group (*P* < .05) see Table 1.

### 3.4. Comparison of the Product of Staining Intensity and Positive Rate of NF-*κ*B Protein

**Figure 5:**
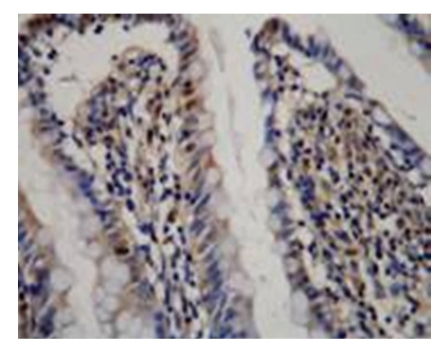
Model control group-28d mucous membrane of small intestine (+++) NF-*κ*B × 200.

**Figure 6:**
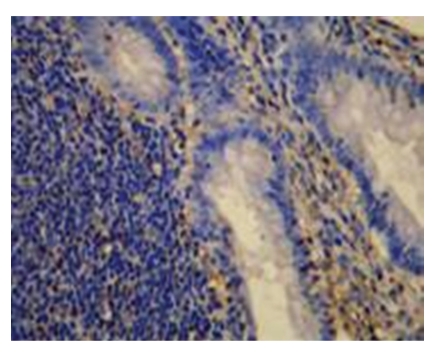
Model control group-28d mucous membrane of small intestine (++) NF-*κ*B × 200.

The positive signals for NF-*κ*B protein were mainly localized in the cytoplasm of intestinal mucosa epithelial cells and occasionally in the cytoplasm of inflammatory cells. The positive signals were mainly localized in the cytoplasm though few were seen in the nucleus. On all time points after operation, no marked difference was noted among all groups (*P* > .05); see Table 1, Figures 5 and 6.

### 3.5. Comparison of the Product of Staining Intensity and Positive Rate of Bax Protein

**Figure 7:**
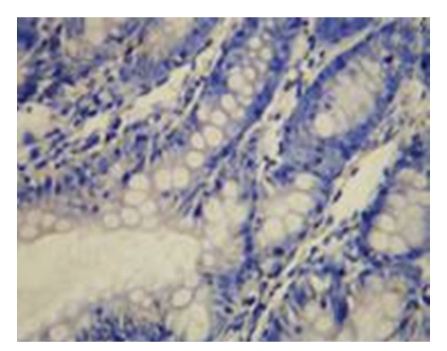
sham-operated group-21d mucous membrane of small intestine (−) Bax × 200.

**Figure 8:**
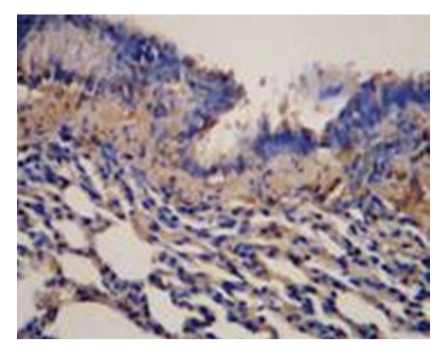
Model control group-21d mucous membrane of small intestine (+++) Bax × 200.

**Figure 9:**
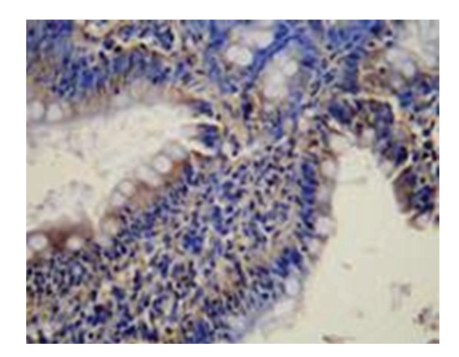
Model control group-21d mucous membrane of small intestine (++) Bax × 200.

**Figure 10:**
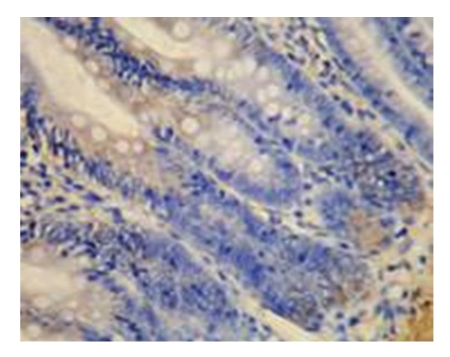
Treated group-21d mucous membrane of small intestine (+) Bax × 200.

The sham-operated group (on 21d) was significantly lower than model control group (*P* < .05); see Table 1, Figures 7, 8, 9, and 10.

### 3.6. Comparison of the Apoptosis Index

**Figure 11:**
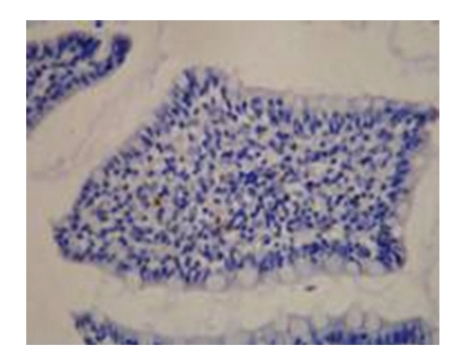
Treated group-21d a few apoptotic cells in mucous membrane of small intestine TUNEL × 200.

The sham-operated group (on 7, 21 and 28d) was significantly lower than model control group (*P* < .05). The treated group (on 14d) was significantly lower than model control group (*P* < .05); see Table 1, Figure 11.

### 3.7. Comparison of Serum MDA

The sham-operated group (on all time points) was significantly lower than model control and treated group (*P* < .01). The treated group (on 14d) was significantly lower than model control group (*P* < .05); see Table 2.

### 3.8. Comparison of Serum SOD

The sham-operated group (on all time points) was significantly higher than model control and treated group (*P* < .05). The treated group (on all time points) was significantly higher than model control group (*P* < .05); see Table 2.

### 3.9. Comparison of Serum NO

The sham-operated group (on 7 and 14d) was significantly lower than model control and treated group (*P* < .01); see Table 2.

## 4. Discussion

The intestine is the largest lymphoid organ of the body. Since the intestinal canal has a constant contact with viruses, bacteria and foreign matters, the surface mucosa of the intestine plays an important role in preventing the invasion of harmful substances. Therefore, protection of small intestinal mucosa is a key to the treatment of OJ. During OJ, high-concentration bile salts and hyperbilirubinemia may be two major factors contributing to the production of oxygen free radicals. Thus, inhibition of hyperbilirubinemia can reduce OJ-induced injury [11, 12]. To a certain extent, MDA is a reliable parameter that can reflect, to a certain extent, oxygen free radical-induced lipid peroxidation and the extent of lipid peroxidation-induced damage to the body. SOD is able to specifically scavenge superoxide anion radicals and thereby exerts some protective effects on the body. This study indicated that the content of serum MDA in astragalus treated group was significantly lower than that in model control group, the content of SOD in astragalus treated group was significantly higher than that in model control group, and the pathological injury of small intestinal mucosa showed varying degrees of mitigation, suggesting that astragalus can reduce the content of serum MDA, increase the content of SOD and mitigate lipid peroxidation-induced cytoplasmic membrane damage, thereby exert protective effects on the small intestinal mucosa. Moderate NO levels are able to expand blood vessels and improve microcirculation, while high levels of NO can aggravate OJ-induced haemodynamic disturbance in the liver and kidney. Han et al. [13] proved that iNOS had an elevated level in the liver of OJ rats and thus could induce the production of a large amount of NO and cause endothelial cell dysfunction. Our results indicated that, though the level of serum NO in astragalus treated group was lower than that in model control group, no statistical difference was observed. We speculate that it may be due to too small sample size.

Nuclear factor *κ*B (NF-*κ*B) is a multieffect transcription regulatory factor that controls the transcription of a variety of inflammation-, immunity-, and hyperplasia-related factors, such as TNF-*α*, IL-1*β*, iNOS, and ICAM-1. Upon activation, NF-*κ*B is able to induce a cascade reaction of cytokines [14] and impair the structure and function of multiple organs [15–18]. Some experimental studies showed that NF-*κ*B may induce or aggravate small intestinal ischemia/reperfusion injury [19], promote intestinal epithelial cell apoptosis [20], and thereby induce intestinal damage. This study showed that at all time points after operation, the expression levels of NF-*κ*B protein showed no significant difference among each group (*P* > .05). So we surmise that NF-*κ*B protein has no significant impact on the pathological injury of the small intestine mucosa of OJ rats.

Apoptosis is a kind of gene-controlled physiological cell death to maintain the body's normal physiological functions and stability. The stability of small intestinal mucosa also depends on the equilibrium between the proliferation and apoptosis of epithelial cells [21]. Some factors such as ischemia/reperfusion injury, bacterial infections and nutritional deficiencies may induce the apoptosis of intestinal epithelial cells [22–27] and cause intestinal dysfunction [28, 29]. Yang et al. [30] found that the total extract of astragalus had inhibitory effects on both in vivo and in vitro hepatic cell injury and apoptosis. In the present study, we found that, on day 14, the apoptosis index in small intestinal mucosa in astragalus treated group was significantly lower than that in model control group, and small intestinal mucosal inflammation and necrosis and microvilli defects in treated group were milder than those in model control group, indicating that astragalus can exert protective effects on the small intestinal mucosa of OJ rats through inhibiting the apoptosis of small intestinal mucosal epithelial cells. Bax, as an apoptosis-inducing gene [31–35], can promote Bcl-2 [36–38] or Bcl-x dimer formation and thus induce cell apoptosis. In this study, no statistical difference existed in the products of the staining intensity and the positive staining rate of Bax protein at all time points was noted between treated group and model control group, suggesting that Bax protein may have no impact on OJ-induced intestinal mucosa injury and other unknown factors are involved in this process.

In summary, astragalus injection can increase the content of serum SOD, decrease the content of MDA, and inhibit the apoptosis of small intestinal mucosa cells, thereby mitigate the pathological injury of the small intestinal mucosa and exerting protective effects on the small intestinal mucosa of OJ rats. However, it is worth mentioning that the therapeutic effects of astragalus are limited and auxiliary. Therefore, combined use of other drugs that can protect the small intestinal mucosa is needed during the course of treatment.

## Figures and Tables

**Table 1:** Comparison of different pathological indexes (*M*(*Q*_*R*_)).

Index	Time	Sham-operated group	Model control group	Treated group
Pathological severity score	7d	0.0 (1.0)	1.0 (1.0)*	0.0 (1.0)*^+^
	14d	0.0 (1.0)	1.0 (2.0)*	1.0 (1.0)^+^
	21d	0.0 (0.0)	2.0 (1.0)*	0.0 (1.0)
	28d	0.0 (0.0)	2.0 (1.0)*	1.0 (0.0)

Product of the staining intensity and positive rate of Bax	7d	0.0 (0.0)	0.0 (1.0)	0.0 (1.0)
	14d	0.0 (1.0)	2.0 (4.0)	0.0 (1.5)
	21d	0.0 (0.0)^+^	1.0 (2.0)	0.0 (1.0)
	28d	0.0 (1.0)	0.5 (1.5)	1.0 (2.0)

Apoptosis index	7d	0.0 (0.0)^+^	0.0 (0.0)	0.0 (0.0)
	14d	0.0 (0.0)	0.0 (0.01)	0.0 (0.0)^+^
	21d	0.0 (0.0)^+^	0.0 (0.01)	0.0 (0.0)
	28d	0.0 (0.0)^+^	0.0 (0.0)	0.0 (0.0)

Product of the staining intensity and positive rate of NF-*κ*B	7d	0.0 (0.0)	0.0 (2.0)	0.0 (2.0)
	14d	0.0 (2.0)	0.0 (4.0)	0.0 (1.0)
	21d	0.0 (0.0)	0.0 (4.0)	0.0 (3.0)
	28d	0.0 (2.0)	1.0 (3.0)	0.0 (1.0)

Note: Compare to sham-operated group, *P < .05 and **P < .01; Compare to model control group, ^+^ *P* < .05 and ^++^ *P* < .01.

**Table 2:** Comparison of different indexes in blood ($\overset{®}{X}\pm S$).

Index	Time	Sham-operated group	Model control group	Treated group
MDA	7d	2.5 ± 1.2	21.6 ± 3.2**	22.8 ± 4.8**
	14d	2.3 ± 1.4	23.4 ± 3.4**	19.6 ± 7.2**^++^
	21d	3.1 ± 1.7	27.1 ± 4.7**	21.0 ± 6.2**
	28d	3.6 ± 1.0	34.0 ± 9.2**	27.0 ± 12.3**

SOD	7d	56.87 ± 12.62	31.27 ± 12.77*	43.61 ± 8.77*^+^
	14d	53.68 ± 9.14	32.16 ± 8.39*	45.28 ± 11.18*^+^
	21d	57.93 ± 12.77	34.25 ± 12.08*	46.38 ± 16.81*^+^
	28d	57.90 ± 7.83	34.95 ± 9.76*	46.88 ± 11.69*^+^

NO	7d	24.0 ± 11.0	36.0 ± 27.0**	30.0 ± 17.0**
	14d	25.0 ± 10.0	47.0 ± 32.0**	37.0 ± 17.0**
	21d	26.0 ± 13.0	48.0 ± 14.0	44.0 ± 16.0
	28d	29.0 ± 8.0	69.0 ± 28.0	50.0 ± 15.0

Note: Compare to sham-operated group, **P < .05 and *P < .01; Compare to model control group, ^+^ *P* < .05 and ^++^ *P* < .01.
